# Automation in ART: Paving the Way for the Future of Infertility Treatment

**DOI:** 10.1007/s43032-022-00941-y

**Published:** 2022-08-03

**Authors:** Kadrina Abdul Latif Abdullah, Tomiris Atazhanova, Alejandro Chavez-Badiola, Sourima Biswas Shivhare

**Affiliations:** 1 Nuffield Department of Women’s & Reproductive Health, University of Oxford, Level 3, Women’s Centre, John Radcliffe Hospital, Oxford, OX3 9DU England; 2 IVF 2.0 Ltd, School of Biosciences, University of Kent, Canterbury Campus, Kent, England; 3 TFP Simply Fertility, W Hanningfield Rd, Great Baddow, Chelmsford, CM2 8HN England; 4 The Centre for Reproductive and Genetic Health, London, UK

## Abstract

In vitro fertilisation (IVF) is estimated to account for the birth of more than nine million babies worldwide, perhaps making it one of the most intriguing as well as commoditised and industrialised modern medical interventions. Nevertheless, most IVF procedures are currently limited by accessibility, affordability and most importantly multistep, labour-intensive, technically challenging processes undertaken by skilled professionals. Therefore, in order to sustain the exponential demand for IVF on one hand, and streamline existing processes on the other, innovation is essential. This may not only effectively manage clinical time but also reduce cost, thereby increasing accessibility, affordability and efficiency. Recent years have seen a diverse range of technologies, some integrated with artificial intelligence, throughout the IVF pathway, which promise personalisation and, at least, partial automation in the not-so-distant future. This review aims to summarise the rapidly evolving state of these innovations in automation, with or without the integration of artificial intelligence, encompassing the patient treatment pathway, gamete/embryo selection, endometrial evaluation and cryopreservation of gametes/embryos. Additionally, it shall highlight the resulting prospective change in the role of IVF professionals and challenges of implementation of some of these technologies, thereby aiming to motivate continued research in this field.

**Graphical abstract:**

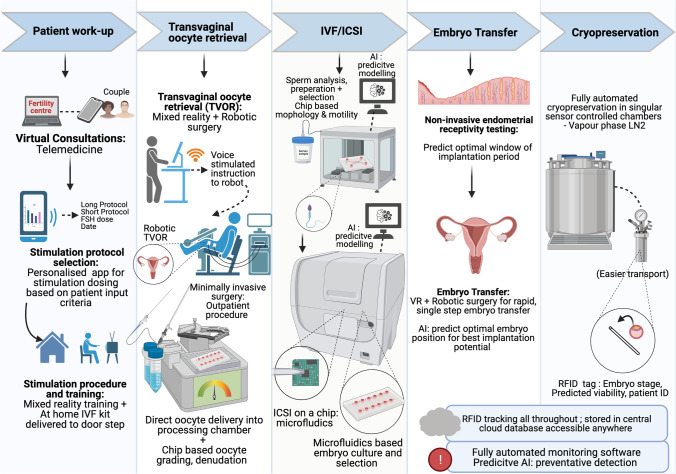

## Introduction

Infertility affects 8–12% of couples worldwide for women aged between 20–44 [1, 2]. The growing safety and popularity of assisted reproductive technology (ART) means that an estimated nine million babies have been born through ART. Strikingly, however, live birth rates in the UK have only marginally improved over the last decade, currently ranging from 31% in patients under 35 to 11% for 40–42 years and over, and 23% live birth for combined age groups [3]. Although live birth rates in the USA remain higher (48–50% in patients under 35 to around 12% for 41–42 years), there is still scope for much improvement [4, 5]. The lack of improvement of overall live birth rates may be attributed to a combination of limitation of current technology and our understanding of the role of age on ovarian reserve and gametes’ quality, in vitro human embryo development, the implantation potential of the human endometrium, and the cumulative effect of each procedural step on success rates of in vitro fertilisation (IVF) treatment. The manual nature of these processes challenges the reproducibility and efficiency of ART. Furthermore, many existing steps in ART are labour- and time-intensive, as well as subject to high inter- and intra-observer variability. Therefore, a significant increase in success rates demands optimisation of each of these steps in an IVF treatment pathway.

In the current age of digitalisation, automation holds a promising role in improving efficiency, reproducibility, and consistency in ART. Automation may be defined as the mechanisation of tasks usually performed by, or even previously impossible for, humans, through a computerised system [6]. In medicine, this has mainly been achieved using robotic systems and microfluidics. Artificial intelligence (AI) on the other hand may aid automation by incorporating memory through learning and can be trained to perform measured actions without human interaction in certain cases. Classification of AI is difficult due to ambiguity of the term; however, it can be broadly divided into three types based on its capability to perform a myriad of tasks: narrow, general, and strong AI [7]. Currently, the world of computer science has only successfully achieved narrow AI, the weakest form of AI [6, 8]. A further subset of AI includes machine learning (ML) and natural language processing (NLP), which have the ability to learn from the input data and even understand human language respectively, without being specifically programmed. This high processing ability resulted in its popularity and applicability. At present, there has been a notable shift from traditional machine learning approaches (e.g., logistic regression, random forest) to more robust deep learning algorithms, such as artificial neural networks (ANN), convolutional neural networks (CNN), and more recently transformer neural network (TNN) [9]. Deep learning uses hierarchical representations to analyse larger data sets with an increasingly diminished need for human involvement and interpretation [8]. As we enter the next generation of rapid biomedical advancements, it is highly probable that automation will be revolutionising ART success rates. This may be achieved through incorporation of automation and AI into several key steps in ART. This review aims to discuss the existing use and improvements produced by automation within the scope of ART and the strengths and limitations of these approaches.

## Automation: the Next Revolution in ART?

### Data Management

Digitalisation of medical records through electronic medical recording has allowed for the generation of large raw patient data sets, which may act as ‘big data’ within ART. A combination of AI, in particular ML and NLP, with big data, presents a powerful and promising tool for data analysis that reduces the need for manual processing. This can aid in the identification of new predictive outcomes and markers for infertility that would not have been identified through traditional statistical modelling [10]. Additionally, storage of data on a central cloud database improves safety of information storage and transferability between medical centres [11]. Automated patient identification has been achieved with the use of electronic witnessing systems and radio frequency identification (RFID) technology. The widely used RI-witness™ system (Research Instrument, CooperSurgical, Denmark) supports the transition from manual barcode identification, which requires at least two embryologists to confirm sample labelling, to automated RFID-based labelling. This greatly decreases IVF workflow time, improves accuracy, and reduces risk of manual error including gamete mismatch [12]. However, errors may still arise during the numerous steps of micromanipulation and handling of gametes/embryos between dishes; a recent advancement promises a unique AI embryo witnessing system utilizing CNN to track and successfully (100%) assign patient specific key to their fresh embryos, thereby allowing traceability at every micro-movement, alongside eliminating mismatch between embryos of different origin [13]. It must be noted, however, that this algorithm was trained with fresh cleavage and blastocyst stage embryos, therefore, not yet applicable for use with frozen embryos. Moreover, the AI algorithm may require further systemic evaluation prior to routine clinical use.

### Patient Treatment Pathway

Integration of AI in the very first steps of ART has allowed for a personalised medicine approach, especially in the characterisation and decision-making process of patient stimulation protocol and dosing, as well as predictive modelling for determination of success. For example, an algorithm designed to predict live birth rates from patient anti-Müllerian hormone (AMH) levels saw high predictive ability with low error rates [14, 15]. Similarly, the PIVET algorithm, aimed to personalise recombinant follicle-stimulating hormone (rFSH) dosing using input patient data such as BMI, age, and antral-follicle counts (AFC) [16]. Furthermore, an individualised rFSH dosing algorithm based on women’s AMH and body weight (Rekovelle®, Ferring Pharmaceuticals Ltd, UK) has been recently shown to reduce risk of OHSS [17]. Such clinical application of prediction outcomes throughout the entirety of IVF has already been used and made commercially available through digital platforms such as Uni*vf*y® (Univfy Inc, USA). Similarly, many fertility units are developing patient-focused mobile applications, with the aim to generate clinically relevant ‘big data’ through the integration of intelligent algorithms. Whilst the potential these applications hold must not be underestimated, their market adoption within IVF may take time; a recurrent issue within the clinical transferability of many automated innovations. Additionally, we must acknowledge that the diversity of examples in a given data set is paramount to the training of an AI algorithm and later generalization of a system. Therefore, the fact that the data currently collected is limited by the size and geographical distribution of the individual or chain of units could become particularly limiting, given, for example, the role of ethnicity in determining current IVF success rates [18].

### Trans-vaginal Oocyte Retrieval

Oocyte retrieval essentially consists of two steps: ultrasound-guided aspiration of follicular fluid (containing oocytes) from the ovary into test tubes followed by microscopic recovery of oocytes in a petri dish in the IVF laboratory. Matsubayashi and colleagues showed that application of AI in recognition of empty and oocyte-containing follicles, trained from ultrasound images, may aid accuracy and decrease the duration of trans-vaginal oocyte retrieval (TVOR), performed by surgeons [19]. Furthermore, the development of a fluid filled, oocyte retrieval chamber (Eggcell) by scientists from Newcastle University in collaboration with Newcastle Hospitals NHS Trust and Labman Automation, currently in early clinical use, provides hope for a semi-automated process of oocyte recovery alongside protecting the oocytes from temperature and pH variations during TVOR (Trial registration ISRCTN: 15,509,950). It should be noted that neither of the above technologies are currently available for direct clinical use and would require further data and clinical trials prior to approval for routine implementation. Nonetheless, training clinicians or embryologists, alone, to efficiently and accurately perform oocyte recovery, identification of cumulus oocyte complex is a long and fallible process. In comparison, coupling oocyte recovery and selection with robotics and microscopy with in-built computational vision and/or AI tools to identify cumulus-oocyte complexes promises to aid this process.

### Oocyte Selection

Oocyte selection does not currently form a part of routine IVF, albeit due to practical and clinical limitations; while the presence of the cumulus mass surrounding an oocyte makes it practically impossible to determine oocyte maturity/quality, selecting oocytes based on morphological parameters may further reduce the already-limited number of oocytes available for treatment. However, stripping the oocyte of the cumulus mass prior to an IVF-intra-cytoplasmic sperm injection (ICSI) procedure opens the possibility to classify oocytes according to their fertilization/blastocyst developmental potential. Image-trained CNN deep learning algorithms such as AIR-O (Artificial Intelligence Ranking system for Oocytes, IVF 2.0 Ltd., UK) [20], VIOLET (Future Fertility, Canada) promises to outperform skilled embryologists in accurately predicting fertilization and blastocyst development rate [21]. Integration of AI in predicting oocyte developmental potential may be beneficial in cases of social freezing, in managing patient expectations, as part of oocyte allocation strategies during egg-donation cycles and to some extent opens doors for future research determining the effect of different stimulation protocols on oocyte quality. Although it may not be utilized as a de-selection tool yet, most significantly due to the limited starting number of oocytes, improvement/incorporation of intelligent oocyte selection tools might also become significant if generation and use of synthetic oocytes does become a necessary reality [22].

### Semen Analysis and Preparation

Male factor contributes to 50% of infertility cases, 80% of which are due to sperm motility [23]. Therefore, robust semen analysis and preparation form an integral part of effective diagnosis and management of infertility. While application of computer-assisted sperm analysis (CASA) for analysing sperm motility has been in use for a long time, an automated version of this software CASAnova was shown to have the ability to accurately classify alterations in sperm motility [24].

Several attempts have also been made to automate semen preparation for treatment, primarily utilising microfluidics, an emerging technology in biomedicine that employs the use of minute volumes of solvents manipulated on a chamber or chip [25]. Furthermore, microfluidic chips allow incorporation of mechanical barriers, which have been suggested to better emulate natural barriers of the female reproductive tract, ensuring only morphologically normal or progressive motile sperm are isolated [26]. A microfluidic chamber, therefore, promises a non-invasive, high-precision, high-throughput, and rapid means of performing semen preparation, further reducing the risk of DNA damage due to traditional density gradient centrifugation methods for sperm sorting [27]. One such example is the FERTILE (Zymot) device (DxNow Inc., Gaithersburg, MD, USA). This single-use filtered chip, with inlet and outlet chambers, connected by a microfluidic channel has shown to allow for better selection of sperm with improved progressive motility and DNA Fragmentation Index [27]. In addition, this partial automation in semen analysis and preparation has the potential to be fully automated with the incorporation of robotics and further integration with AI, whereby computational modelling may be able to optimise fluid flow and displacement further personalising the process. A close example is the AI and robotics-powered microscopy system, the Mojo©-AISA (Mojo, Sweden), which promises to perform rapid analysis of raw semen samples within ten minutes, compared to the usual 30 min by trained andrologists. The system has been noted to conform to the 2010 World Health Organization (WHO) semen analysis values and shows a promising way of standardising semen analysis which is currently limited by high inter- and intra- observer variation [28]. However, further clinical training of the CNN-based AI algorithm used will be required to reduce the observed high false positive prediction rates, especially significant for low concentration samples.

### Insemination and Intra-cytoplasmic Sperm Injection (ICSI)

The possibility of automating insemination via conventional IVF or ICSI is perhaps quite futuristic and exciting. Nonetheless, attempts have already been made, albeit strictly for research purposes and in animal models, to automate conventional IVF utilising microfluidic chips [29, 30]. For example, Han et al. designed a two-layered, 200-µm microwell array microfluidic device for the single-step trapping and in situ insemination of oocyte with sperm as well as simultaneous embryo culture [29]. Insemination using conventional IVF essentially consists of two steps: sperm preparation and introduction of a specific concentration of sperm to an oocyte; therefore, this may seem less challenging to automate.

Contrastingly, insemination using ICSI is a multi-step process: oocyte denudation, oocyte selection based on maturity, sperm immobilisation, holding and injection needle positioning, oocyte positioning, zona and oolemma rupture and finally injection of the sperm. Thus, ICSI is far more complex, challenging, time-consuming, and consequently strictly operator dependent. Despite these challenges, attempts have been made to automate several of these steps. A microfluidic chip-based system successfully automated the denudation of oocytes and oocyte selection based on oocyte sedimentation rate prior to ICSI. Good-quality oocytes settled in lower outlet chambers as proven by cytoplasmic maturity and blastocyst formation rates [31, 32]. Another contact-free oocyte denudation process has been presented recently, which proposes the use of ultrasonic waves to subsequently denude oocytes by induced acoustic streaming and acoustic radiation force, within a microfluidic device [33]. Oocyte positioning to avoid the polar body during ICSI is normally performed manually. A Hough transform algorithm trained with input oocyte images has been shown to successfully detect 100% of polar body location in various images of different magnification and background, allowing use of this algorithm across different microscopes [34]. This type of AI-based oocyte positioning may perhaps be integrated into existing imaging systems such as the Oosight® (Hamilton Thorne, USA), which uses liquid crystal technology to visualise oocyte spindle, thereby aiding oocyte positioning prior to ICSI.

Attempts have also been made to automate sperm immobilisation [35, 36]; integration of automation using a robotic arm and a visual servo control AI algorithm in sperm tracking and immobilisation prior to ICSI showed 96% and 94.5% success rates, respectively [36]. However, this system requires initial operator selection of sperm based on morphology, which makes it subjective. Another promising design, FertDish, combines motile sperm selection directly using channels and transfer capillaries in one dish and shows high clinical feasibility as it is adapted onto existing clinically used ICSI dishes [37]. While the above highlight the scope for partial automation for sperm selection and immobilisation, an example of the use of AI algorithm for sperm selection with the aim for improved ICSI outcome and subsequent embryo development would be the SiD software (V1.0; IVF 2.0 Ltd.) [38]. Though promising, the study demands larger data sets, while caution should be taken during gauging future implications since oocyte competence and operatory competency are very likely to have a direct effect on the outcome of ICSI and potential embryo development.

Finally, robotic injection of sperm showed a 90% success rate, performed in comparable times to experienced operators [39]. Interestingly, the rise in electrical resistance following oolemma penetration was suggested to be used as a measure of penetration success using an electrical adaptor system connected to the injecting needle [40]. The scope for an automated sperm injection robot functioning with the help of AI algorithms, optics, cell microinjectors and mechatronics, has recently been presented, such that their efficiency with respect to injection time, survival, cleavage, and blastocyst development rate in mouse and hamster gametes/embryos, were comparable to manual controls [41]. The main limitations of many of these novel approaches described here are in the use of animal models which are different in terms of gamete size, cytoplasmic integrity, and resistance compared to human gametes. Although this questions the reliability and transferability of these systems in a clinical setting; the only way forward is further research using human gametes. While clinical implementation of automated ICSI may be a futuristic dream, improving existing ICSI methodology with the help of AI algorithms may not be, where a trained AI model is able to standardise as well as optimise ICSI procedures, resulting in better clinical outcome [42, 43]. However, these models are at their nascent developmental stage and would require further data and training prior to clinical implementation.

### Embryo Culture and Selection

In the recent past, the development of single-step continuous culture medium to grow a zygote to blastocyst stage has paved the way for the use of time-lapse imaging (TLI) incubators such as the Embryoscope™ (Vitrolife, Sweden) and Geri® (Merck group, Germany) in human IVF laboratory. This, in turn, has allowed continuous automated monitoring of embryo development without exposure to environmental stress. Although multiple studies have shown improvement in success rates using Embryoscope, results remain varied between laboratories [44]. Additionally, the TLI culture consists of two elements: continuous undisturbed culture and monitoring of morphokinetic parameters, which a multi-centre randomised clinical trial is aiming to dissect in conjunction with clinical outcome [45].

Nonetheless, clinical implementation of the TLI culture system has allowed for an exponential evolution in embryo grading from conventional morphology grading to morphokinetic grading and finally integration of AI algorithms, a step towards automation [46–52]. Multiple studies investigating the potential benefit of multi-time point evaluation of morphokinetic parameters in embryos resulted in the growing interest and application of TLI and morphokinetic embryo grading [51, 53–58]. Consequently, embryo selection is currently based on a combination of morphological and kinetic parameters often utilising semi/automated and/or AI algorithms such as KIDScore® (Vitrolife, Sweden) and Eeva® System (Merck group, Germany). However, TLI systems are expensive, some require manual annotation of key parameters by embryologists, and thus are still subject to inter-observer variability [59]. AI has been introduced to reduce human decision-making involved in TLI through the integration of algorithms such as the iDAScore® system (Vitrolife, Sweden). Other AI algorithms exist; STORK, based on Google’s Inception model, has been indicated to predict blastocyst quality with an AUC of > 0.98 outperforming embryologists [60]. Contrasting to the KIDScore®, iDAScore®, and Eeva® systems which utilise time-lapse images, other promising AI embryo selection tools such as AIR-E (Artificial Intelligence Ranking system for Embryos, IVF 2.0 Ltd., UK) and Life Whisperer© (Presagen, Australia), both computer-based softwares, provide deep learning platforms using static images of embryos, requiring no costly equipment, making its clinical application most affordable and flexible [61, 62]. Most of the afore-mentioned algorithms are currently in clinical use with limitations; while it is impossible to achieve a 100% implantation prediction potential, the use of AI in doing so can only increase the probability of successful implantation.

### Preimplantation Genetic Testing and Metabolomics

Despite the conflicting evidence of the effectiveness of preimplantation genetic testing (PGT) in improving ART success rates [63–67], it is widely used as an embryo selection tool in many IVF clinics [65]. This growing demand for PGT has led to the development of AI integrated platforms such as PGTai^SM^ (CooperSurgical, Denmark), a predictor algorithm that increases the sensitivity, efficiency, and objectivity of PGT-aneuploidy (PGT-A) sequencing data analysis by reducing human involvement in the process. Excitingly, to circumvent invasiveness of PGT-A altogether, image-based detection of embryo ploidy and prediction of embryo success rates at any developmental stage has been explored through the creation of an AI algorithm, ERICA® (Embryo Ranking Intelligent Classification Algorithm). ERICA® has been shown to be superior in its ability to predict blastocyst ploidy status and selection of embryos with best clinical outcome with a 92.5% success rate, when compared with trained embryologists [68]. Additionally, this dynamic ERICA® algorithm has recently been tested for its ability to be personalised according to individual clinic protocols and procedures [69] and a positive correlation has also been shown between lower ERICA® grades and chances of early miscarriage, independent of patient age [70]. However, this algorithm would require further development using diverse training data sets.

A non-invasive approach to PGT-A (NIPGT) has been shown to accurately test for ploidy status utilising cell-free DNA within spent culture system, with striking concordance to routine invasive trophectoderm biopsy [71]. Interestingly, a non-invasive metabolomic approach has been used in the design of a predictive algorithm based on 60 potential biomarkers of embryo aneuploidy, discovered through spent culture media analysis. The algorithm showed a 97.5% accuracy rate in selecting aneuploid over euploid embryos [72]. This approach of using AI for non-invasive embryo selection has been commercialised by the Overture© Metabolomics (Overture Life, USA). Additionally, Raman spectroscopy–based metabolic profiling combined with AI for ploidy prediction has also been demonstrated [73]. It is evident that there is scope for embryo selection based on its ploidy status; what remains to be seen, however, is if/how we may be able to harness the combined strength of NIPGT and metabolomics through AI algorithms to significantly improve clinical outcomes, thus making them undeniably cost effective.

### Endometrial Evaluation for Personalised Embryo Transfer

Advancements in molecular techniques have more recently allowed detailed study of endometrial receptivity [74–77] resulting in a genomic diagnostic tool, endometrial receptivity array (ERA) [78], and subsequent personalised embryo transfer (pET). The ERA utilises endometrial gene expression analysis, which, integrated with AI, aims to increase accuracy and reproducibility when compared with conventional histological analysis of the endometrium [79]. Although ERA continues to be used clinically, there is conflicting evidence on its clinical benefit [80–85].

### Cryopreservation and Cryostorage

The first automated cryopreservation (vitrification) device, Gavi® (Genea Biomedx, Australia), showed equivalent clinical efficiency in embryo survival post-embryo thaw when compared to existing manual methods [86]. The automated equilibration of embryos prior to vitrification offers reduced labour intensity and improved accuracy with the maintenance of optimal conditions such as embryo positioning, cryoprotectant concentration, exposure time, cooling rates, volume, and temperature [87]. Another cryopreservation system, Sarah® (FertileSafe Ltd, Israel), along with the warming system Helia® (FertileSafe Ltd, Israel), uses a robotic arm for rapid exposure of embryos in straws into equilibration and vitrification solutions and finally into liquid nitrogen. This device was shown to produce 100% and 95% survival, for embryos and oocytes, respectively. However, further device validation is required prior to clinical use, since validation studies were performed using an animal model [88]. Additionally, both of the systems are semi-automated, costly, and still require considerable human interaction for operation, thus limiting routine clinical implementation of automated cryopreservation systems.

Cryostorage, post cryopreservation presents us with several short- and long-term challenges such as risk of specimen contamination, reduction or loss of viability, loss during shipping, and risks of shipping and handling [89]. Although legal principles and best practices exist to minimise such losses [90], they may never be completely eliminated. However, recent advancements in the automated management of cryopreserved gamete and embryo storage through a data management system introduced by TMRW® Lifesciences gives a much needed scope for improvement [91, 92]. The ivFOS® system integrates RFID technology and centralised cloud database storage to ensure unique patient identification of all cryopreserved samples, accessible from any screen in the world. The use of AI and automation in the maintenance and monitoring of critical parameters and conditions in the cryopreservation tanks has also been achieved through their TMRW overwatch™ system, which is integrated with its own algorithm for early prediction of future system failures.

## Painting a Realistic Future

### Limitations and Clinical Feasibility

Implementation of any novel technology is challenging. Despite the breadth of efforts to automate the procedural steps of ART, there has been relatively small penetration into the clinic due to practical restrictions, ethical concerns, and importantly lack of further research and clinical trials.

This review highlighted a notable shift in ART, towards utilisation of a combination of automated and/or more independent self-supervised deep learning algorithms with higher processing potential and reduced human bias. However, algorithmic bias may still be an overarching limitation of such black box modelling, resulting from self-supervised applications. Supervised learning is fundamentally biased and could easily become resource-intensive in terms of computational power and human resources, making the automation of such process desirable. One potential solution is the self-supervised learning approach, as is the case with the use of adversarial networks (AANNs)s, where a second AI is programmed to evaluate and try to outperform the original one. An example of AANNs successfully tested in the field of reproductive medicine was presented by Kanakasabapathy et. al., where they subjected an AANNs to evaluate embryos, sperm, and blood cells using a range of images from different image qualities [93]. Although the current clinical value of this approach is still to be tested, the authors make an interesting proposal towards generalisation and automation of an AI system. In a clinical setting, the safety and outcome of utilising a predictive algorithm based on what cannot be fully understood by a healthcare practitioner can be both questionable and detrimental, thereby necessitating regular systematic performance assessments [94]. Additionally, we need to understandably acknowledge that AI in its current form (i.e., narrow AI) is better approached as an assisting tool, rather than as a replacement, for embryologists and clinicians, and must only be implemented through well-designed researched processes. Moreover, a combination of an expert human, a machine, and a well-designed process is highly likely to outperform either machine or human, alone [95]; such combined approach may circumvent the possibility of lack of control over clinical decision-making going against the innate human nature of an experienced embryologist.

Another rate-limiting step is practicality and importantly the question of cost-effectiveness. For example, the semi-automated Gavi® cryopreservation system is much more costly compared to manual cryopreservation, for the same blastocyst survival rates post thawing. This is reflected in the poor clinical use of the system, where, despite this novel technique being available since 2013, live birth rates were only recorded by 2017 in Europe [96].

Usability and integrability of many of these automated approaches are also limited in certain cases, such as the Gavi® system. This system operates with Gavi® only consumables rather than existing consumables, further limiting its accessibility while also increasing its cost. Similarly, microfluidic approaches, though promising for semen analysis and sperm preparation, may be less feasible in high-throughput laboratories, due to the necessity of a high volume of raw semen assessment requiring multiple chips.

Lack of robust clinical evaluation through randomised control trials (RCT) to support the safety and efficacy of many of these automated and AI systems immensely restricts much-needed evidence for/against clinical use as well as highlights the need for further clinical research. This lack of quality in evidence may be attributed to the time from ideation to publication of results from an RCT, data insufficiency, variability in data sets, and bias within individual studies [19, 94]. Again, bias greatly limits transferability of study approaches between groups due to inter-clinic variation including niche laboratory conditions and heterogeneous data points such as different input and output measures [97]. Data insufficiency, particularly prospective data, limits the use of large and representative data sets in many of these studies. These issues restrict the reliability and reproducibility of AI, which is essential prior to generalised clinical use [98]. While the literature remains conflicted in terms of an ‘ideal’ size for data sets, usage of synthetic data, albeit arguable, has been suggested as a probable approach to overcoming such shortcomings [99]. This is especially true in the UK, where ART is regulated by the Human Fertilisation and Embryology Authority (HFEA), and all products used in an IVF laboratory must be CE marked prior to clinical use, requiring evidence of effectiveness backed up by large RCTs. Blockchain technology, which offers a decentralised and freely available breadth of prospective anonymous patient data, has been proposed to overcome these issues and could greatly increase the strength of generalised AI use clinically [98].

As with many initial advancements and especially within IVF, ethical regulations must be addressed. With the high processing power of big data brought about using general processing units (GPUs) in AI, it is important to have Health Insurance Portability and Accountability Act (HIPAA)–compliant patient data protection software in place and perhaps the development and implementation of AI-driven defence mechanisms [99]. Furthermore, user transparency and responsible disclosure systems must also be in place prior to clinical use of AI systems [11].

### Future of ART

As we enter the era of Web 3.0, exponential advancements in medical sciences have followed due to integration of computer and biomedical sciences. The researched areas reviewed here together with future extrapolations from other fields such as soft robotics and telesurgery [100] may result in an ART process that is very much distinct from what it is today. Mixed reality (MR), combining virtual reality (VR) and augmented reality (AR), may be an option to fully automate patient consultations especially significant in times of global pandemics. Operative procedures such as TVOR and embryo transfer may also be performed with mixed reality, the main benefit of which removes physical limitations of consultants to one clinic [101]. Synergy between microfluidics [102], AI, and robotics may indeed achieve a fully automated, intelligent, single-step device for the entirety IVF treatment pathway. This idea of ‘IVF in a box’ has already been conceptualised by NaturaLife (Overture Life, USA), which currently offers three limited features including cryopreservation of oocytes and embryos and non-invasive testing of embryos.

## Conclusion

In conclusion, as the world is at the forefront of science and technology, implementation of novel technologies for the automation of ART will soon become a reality. The goal of automation in ART is not to replace the role of embryologists and practitioners but rather to aid in its improvement. In addition, considering the foreseeable increase in demands of ART due to a rise in infertility cases, popularity of social freezing and the pursuit of ‘healthy’ offspring, in the future, the number of embryologists may not be reduced, but their role may change considerably from performing repetitive mechanical tasks to precise logical decision-making. Indeed, it would be interesting to foresee, if, in fact, today’s clinical embryologists may re-evolve into at least partial research embryologists in the near future. The benefits of automation in the future of ART are clear; standardisation and increased precision to increase efficiency and accessibility of ART will become a necessity. However, taking into consideration the minimal turnover of concepts into commercially available clinical applications, the gap between research and clinical implementation of innovative technologies must first be bridged in order to standardise the use of automation and AI in improving ART.

## Data Availability

Not applicable.
